# Imaging peculiarities of gubernaculum tracts in molars as accessional teeth on CT

**DOI:** 10.1002/cre2.452

**Published:** 2021-06-17

**Authors:** Masafumi Oda, Ikuko Nishida, Manabu Habu, Osamu Takahashi, Shirou Tabe, Hiroki Tsurushima, Taishi Otani, Daigo Yoshiga, Teppei Sago, Tatsurou Tanaka, Nao Wakasugi‐Sato, Shinobu Matsumoto‐Takeda, Masaaki Sasaguri, Yasuhiro Morimoto

**Affiliations:** ^1^ Division of Oral and Maxillofacial Radiology Kyushu Dental University Kitakyushu Japan; ^2^ Division of Developmental Stomatognathic Function Science Kyushu Dental University Kitakyushu Japan; ^3^ Division of Maxillofacial Surgery Kyushu Dental University Kitakyushu Japan; ^4^ Division of Oral Medicine Kyushu Dental University Kitakyushu Japan; ^5^ Division of Dental Anesthesiology Kyushu Dental University Kitakyushu Japan

**Keywords:** accessional tooth, CT, gubernaculum tract

## Abstract

**Objectives:**

The shapes of gubernaculum tracts (GTs) in molars as accessional teeth remain unidentified. To elucidate imaging peculiarities of GTs in molars with aging on multidetector‐row computed tomography (MDCT).

**Material and methods:**

This retrospective study was conducted using CT images, including maxillary and mandibular molars, with no abnormal findings from 239 patients. Shapes of alveolar bone, GTs, and dental sacs of the maxillary and mandibular molars were analyzed multi‐sectionally. Correlations between 2‐ and 3‐dimensional imaging figures of GTs in molars and chronological age or stage of molar formation were analyzed.

**Results:**

Some forms of GTs in maxillary and mandibular third molars were observed. In the early stage, GTs were visualized as bone defect lines on the dentition and grooves on the mesial alveolar crest continuous with the dental sac to mesial tooth bud. GTs of the third molar formed a J‐shape in maxillary teeth and Y‐shape in mandibular teeth in the middle stage, as alveolar bone around the GT developed. In the mature stage, the course of the GT changed to straight and perpendicular. Some GT forms were also identified in first and second molars. Significant correlations were found between GT alterations and chronological age or stage of molar formation. Moreover, tracts continuing from the distal side of mandibular third molars were detected.

**Conclusions:**

This paper describes the peculiarities and process of progression for GTs in molars, and the existence of tracts continuing from the distal side of mandibular third molars, unlikely dentition with deciduous predecessors. These preliminary data should prove beneficial for studies focusing on GTs in molars.

## 1 INTRODUCTION

The gubernaculum tract (GT) connects the pericoronal follicular tissue of a tooth with the overlying gingiva, constituting a pathway from the dental sac to the gingiva for the eruption of permanent teeth (Cahill et al., 1988; Hodson, 1971; Scott, 1948). The main roles of GTs are likely to involve the induction of normal tooth eruption (Ferreira et al., 2013; Hodson, 1971; Scott, 1948). Our previous reports have paid attention to various characteristics of GTs, elucidated using cone‐beam computed tomography (CBCT) and multidetector‐row CT (MDCT) (Nishida et al., 2015; Oda et al., 2018; Oda, Miyamoto, et al., 2016; Oda, Nishida, et al., 2016). In those studies, GTs of the maxillary and mandibular molars were visualized as narrow, round‐shaped, hypodense areas over unerupted molars on axial CT. In our advanced analyses of the imaging characteristics of GTs, we noted the possibility of imaging peculiarities for the GTs of accessional teeth and their alterations with aging. In the past, arguments have been made both for and against the existence of GTs in accessional teeth (Cahill et al., 1988; Cahill & Marks Jr., 1980; Ferreira et al., 2013; Gaeta‐Araujo et al., 2019; Hodson, 1971; Ide et al., 2011; Koc et al., 2019; Philipsen et al., 1992; Scott, 1948). However, recent studies have confirmed the existence of GTs in accessional teeth and the relationships between status of GTs and abnormal eruption (Gaeta‐Araujo et al., 2019; Koc et al., 2019). Confirming the shapes, course, and alterations of GTs in molar is thus increasingly important.

However, no studies appear to have investigated such imaging peculiarities and their alteration with aging in accessional teeth. To evaluate this possibility, the imaging characteristics and alteration of GTs in the first, second, and third molars were precisely and retrospectively analyzed according to aging using MDCT.

## 2 MATERIAL AND METHODS

This retrospective study was conducted using CT images from 239 patients (120 males, 119 females; mean age, 11.5 years; range, 1–28 years) obtained in the Division of Oral and Maxillofacial Radiology at Kyushu Dental University Hospital between 2011 and 2020. The criterion of molars for inclusion was visualization of unerupted tooth and surrounding alveolar crest. Low‐quality images with blurring or metal artifacts interfering with the view of GTs were excluded. The numbers of maxillary and mandibular accessional teeth enrolled in the present study are shown in Table 1. The present study was approved by the institutional review board of Kyushu Dental University (approval no. 14–29). The need to obtain informed consent for participation in this trial was waived by the review board based on the retrospective design of the study.

**TABLE 1:** Distributions of number of maxillary and mandibular molars enrolled in present study

	Number of maxillary teeth			Number of mandibular teeth		
Age	First molar	Second molar	Third molar	First molar	Second molar	Third molar
1	2	–	–	2	2	–
2	2	–	–	2	2	–
3	4	4	–	4	4	–
4	12	14	–	11	14	2
5	26	40	–	22	34	25
6	2	16	–	2	14	12
7	2	26	2	2	28	22
8	–	28	12	1	32	32
9	–	38	26	–	42	48
10	–	29	31	–	22	35
11	–	8	17	–	4	19
12	–	9	17	–	5	18
13–20	–	3	99	–	4	98
21–30	–	–	12	–	–	10
Total	50	215	216	46	207	321

CT examinations were performed on 16‐detector‐row CT scanners (Activion 16 system; Toshiba Co., Tokyo, Japan) with 1 s/rotation, and 0.3‐mm‐thick images were reconstructed using bone algorithms in the axial plane to examine each region within the maxilla and/or mandible. All CT images were analyzed using a PC‐based digital viewing system (Ziostation 2; Ziosoft, Tokyo, Japan). This analysis was performed with multiplanar reconstruction methods that allow the selection of appropriate slices for visualizing GTs and for adaptation of the level of the gray scale in the images. In addition, 3‐dimensional (3D) images of GTs in the accessional tooth were produced and analyzed in respective subjects using CT datasets.

The shapes of alveolar bone, GTs, dental sacs, and level of calcification of teeth were analyzed on 2‐dimensional (2D) images in a multi‐sectional manner. The 3D images were then used to identify positional relationships among them and the course of GTs. Both 2D and 3D images of GTs in accessional teeth were also evaluated according to age. At the same time, molars were divided into seven groups based on the stages of tooth formation. Stages were as follows: not calcified, no calcification of the crown was observed; cusp calcifying, only cusps of the crowns were calcified; crown calcifying, calcification of the crowns was not completed; crown formation completed, the entire crown formation was completed and root formation was less than the furcation level; root developing, development of root height had not yet ended; root apex not closed, development of the root height had ended, but the apical end was still open; and root formation completed, the apical end of the root was closed. Figures of GTs in accessional teeth were evaluated according to the stages of tooth formation. Images were assessed by a single oral and maxillofacial radiologist (M.O.) with 14 years of experience.

SPSS version 11 statistical software (SPSS, Chicago, IL) was used for all statistical analyses. Pearson's correlation coefficient was used to compare categorical variables. Values of *p* < 0.05 were considered significant.

## 3 RESULTS

### 3.1 Visualization of GTs in maxillary accessional teeth on CT

**FIGURE 1:**
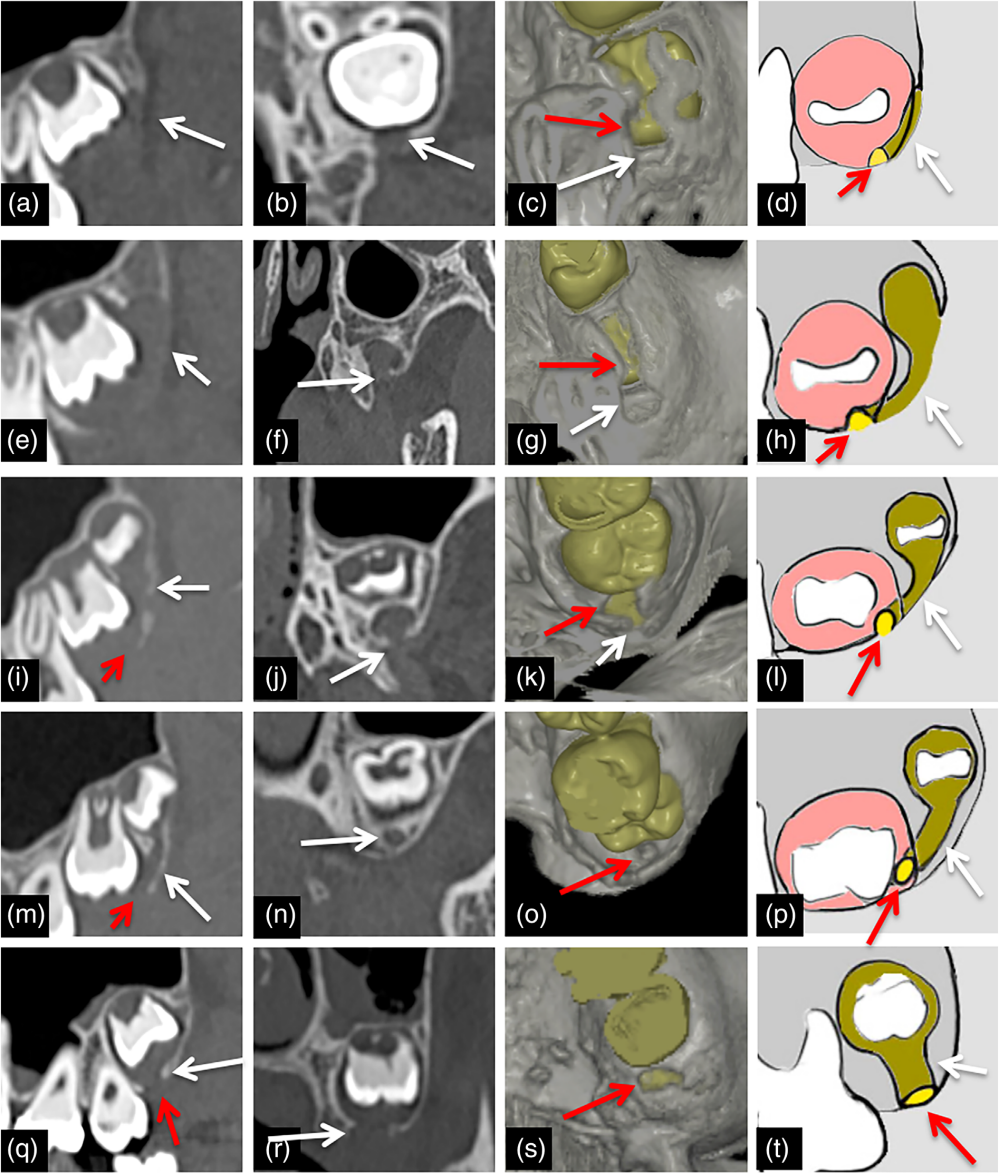
Representative images of GTs for the maxillary molar of each form. (a)–(d) Representative sagittal (a) and axial (b) images, 3D‐CT image (c) and schema (d) of the sprouting‐form GT in maxillary molar. GTs are visualized as bone defect lines (arrows) on the distal alveolar crest continuous from the top of the mesial dental sac (red arrows). (e)–(h) Representative sagittal (e) and axial (f) images, 3D‐CT image (g) and schema (h) of the groove‐form GT in maxillary molar. GTs are visualized as bone defect lines consisting of thin bone defect area on the dentition (arrows) and groove on the mesial alveolar crest continuous from dental sac to mesial tooth bud (red arrows). (i)–(l) Representative sagittal (i) and coronal (j) images, 3D‐CT image (K) and schema (L) of imperfect‐tubular‐form GT in maxillary molar. GTs are visualized as bone defect lines consisting of thin bone defect area on the tooth (arrows) and imperfectly tubular‐shaped bone defect on mesial alveolar crest (red arrows). (m)–(p) Representative sagittal (m), coronal (n) images, 3D‐CT image (o) and schema (p) of tubular‐form GT in maxillary molar. GTs are visualized as bone defect lines consisting of thin bone defect area on the tooth (arrows) and formation of perfectly tubular‐shaped bone defect on the mesial alveolar crest (red arrows). GTs are visualized as showing a J‐shape. (q)–(t) Representative sagittal (q) and coronal (r) images, 3D‐CT image (s) and schema (t) of hole‐form GT in maxillary molar. GTs are visualized as bone defect lines (arrows) that run perpendicular from the dental sac to the alveolar crest (red arrows)

Representative 2D and 3D images of GTs in the maxillary accessional teeth of subjects are shown in Figure 1.

In a very early stage, such as the uncalcified stage of maxillary third molars, GTs were visualized as bone defect lines on the distal alveolar crest continuous from the top of the dental sac (Figure 1(a)–(d)). These GTs were termed “sprouting form” based on the particular findings.

In an early stage, such as the crown‐calcifying stage of maxillary first molars, the cusp‐calcifying stage of maxillary second molars, and the uncalcified stage of third molars, GTs were visualized as bone defect lines consisting of a thin bone defect area on the dentition and grooves on the mesial alveolar crest continuous with the dental sac to mesial tooth bud (Figure 1(e)–(h)). These GTs were termed “groove form” based on the particular findings.

In maxillary second and third molars, however, GTs in a middle stage, such as the cusp‐calcifying stage, displayed a characteristic feature. These GTs were visualized as bone defect lines comprising thin bone defect areas on the tooth and an imperfect tubular‐shaped bone defect on the mesial alveolar crest (Figure 1(i)–(l)). These GTs were termed “imperfect‐tubular form” based on the particular findings. In addition, in third molars, such as those in cusp‐calcifying periods, GTs formed a perfect tubular form and were visualized as a J‐shape (Figure 1(m)–(p)). The structures were initially perpendicular to occlusal plane parts near the dental sac, and then bent mesially to become parallel. The parallel part directed mesially joined the top of the mesial dental sac. These GTs were termed “tubular form” based on the particular findings.

In a late stage, such as the whole crown calcified stage of maxillary first, second, and third molars, GTs were visualized as bone defect lines that ran perpendicularly from the dental sac to the alveolar crest (Figure 1(q)–(t)). These GTs were termed “hole form” based on the particular findings.

Four cases without GTs were detected for maxillary third molars. These tooth buds were completely surrounded by bone.

Detection rates for GTs in the maxillary accessional teeth of subjects are shown in Table 2.

**TABLE 2:** Distribution of forms of GTs in maxillary and mandibular molars and mean age

	First molar			Second molar			Third molar		
Form of GT	Number of cases	Detection ratio (%)	Age(mean ± SD)	Number of cases	Detection ratio (%)	Age(mean ± SD)	Number of cases	Detection ratio (%)	Age(mean ± SD)
Maxillary									
Sprouting	–	–	–	–	–	–	7	3.1	8.3 ± 0.76
Groove	4	8	1.5 ± 0.58	27	12.6	4.7 ± 1.30	6	2.7	8.8 ± 1.17
Imperfect‐tubular	–	–	–	71	33	6.3 ± 1.68	63	28	9.4 ± 1.16
Tubular	–	–	–	–	–	–	9	4	11.1 ± 1.36
Hole	46	92	4.7 ± 0.87	117	54.4	9.1 ± 1.72	136	60.4	14.7 ± 3.44
Not detected	–	–	–	–	–	–	4	1.8	17.3 ± 3.30
Mandibular									
Sprouting	–	–	–	–	–	–	43	13.4	5.8 ± 1.41
Groove	6	13	2.7 ± 1.86	61	29.5	5.3 ± 2.12	129	40.2	9.0 ± 1.67
Imperfect‐tubular	–	–	–	51	24.6	7.8 ± 1.84	16	5	10.4 ± 1.21
Tubular	–	–	–	–	–	–	49	15.3	11.9 ± 2.40
Hole	40	87	4.8 ± 1.10	95	45.9	8.6 ± 1.83	83	25.9	15.9 ± 3.72
Not detected	–	–	–	–	–	–	1	0.3	17.0 ± 0.00

### 3.2 Visualizations of GTs in mandibular accessional teeth on CT

**FIGURE 2:**
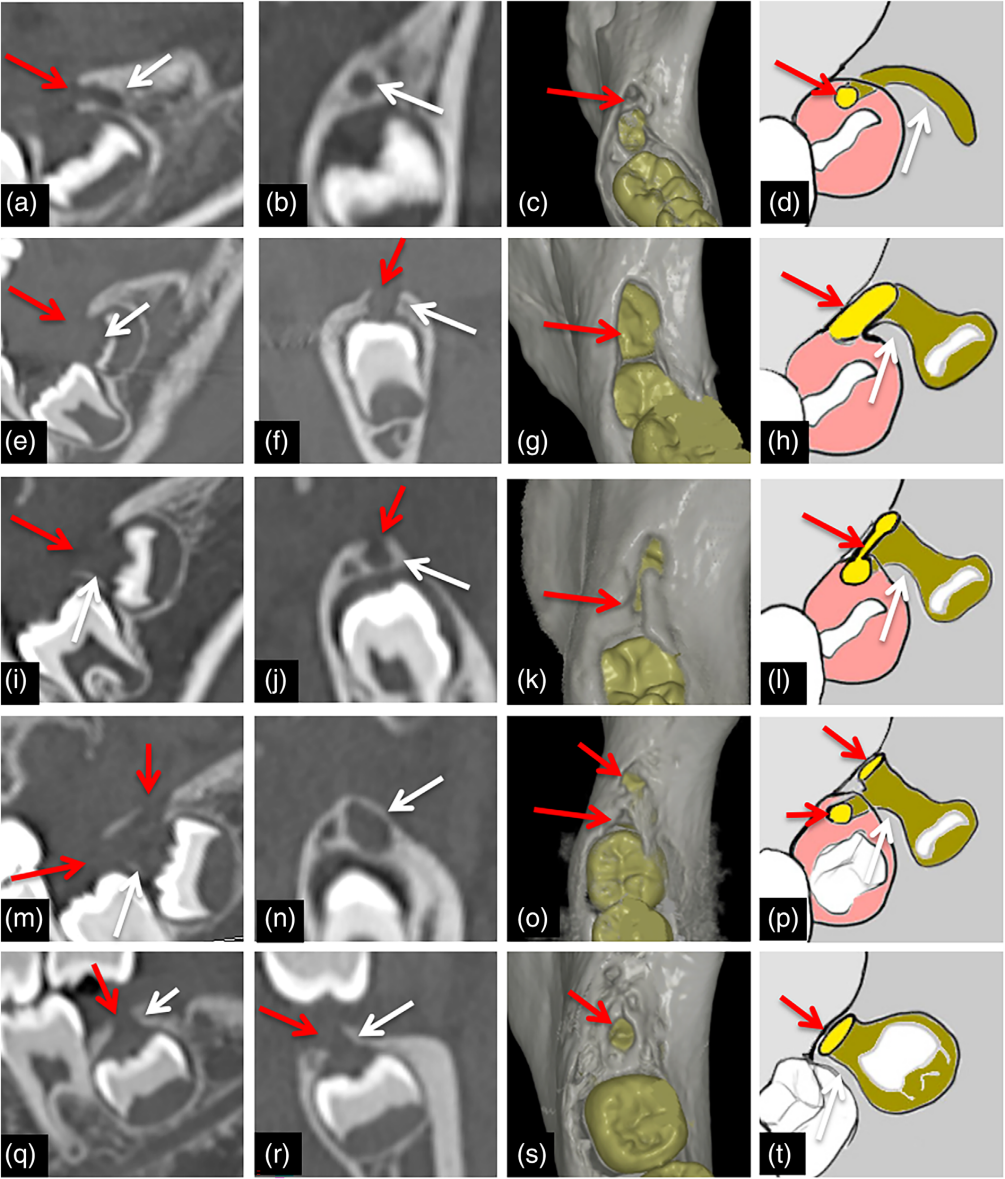
Representative images of GTs for the mandibular molar of each form. (a)–(d) Representative sagittal (a) and coronal (b) images, 3D‐CT image (c) and schema (d) of sprouting‐form GT in mandibular molar. GTs are visualized as bone defect lines (arrows) on the distal alveolar crest continuous from the top of the mesial dental sac (red arrows). (e)–(h) Representative sagittal (e) and coronal (f) images, 3D‐CT image (g) and schema (h) of groove‐form GT in mandibular molar. GTs are visualized as bone defect lines consisting of thin bone defect area on dentition (arrows) and groove on the mesial alveolar crest (red arrows). (i)–(l) Representative sagittal (i) and coronal (j) images, 3D‐CT image (k) and schema (l) of imperfect‐tubular‐form GT in mandibular molar. GTs are visualized as bone defect lines consisting of thin bone defect area on the tooth (arrows) and imperfectly tubular‐shaped bone defect on the mesial alveolar crest (red arrows). (m)–(p) Representative sagittal (m) and coronal (n) images, 3D‐CT image (o) and schema (p) of tubular‐form GT in mandibular molar. GTs are visualized as bone defect lines consisting of thin bone defect area on the tooth (arrows) and formation of perfectly tubular‐shaped bone defect as parallel parts directed mesially (red arrows), and perpendicular parts directed vertically to the alveolar crest (arrowheads). GTs are visualized as showing a Y‐shape. (q)–(t) Representative sagittal (q) and coronal (r) images, 3D‐CT image (s) and schema (t) of hole‐form GT in mandibular molar. GTs are visualized as bone defect lines (arrows) that run perpendicular from the dental sac to the alveolar crest (red arrows)

Representative 2D and 3D images of GTs in accessional dentitions of subjects are shown in Figure 2.

In a very early stage, such as the uncalcified stage of mandibular third molars, GTs were visualized as sprouting forms, similar to the maxillary accessional dentition forms (Figure 2(a)–(d)). In an early stage, such as the crown‐calcifying stage of mandibular first molars, cusp‐calcifying stage of mandibular second molars, and uncalcified stage of third molars, GTs were visualized as groove forms, similar to maxillary accessional tooth forms (Figure 2(e)–(h)). In mandibular second and third molars, GTs in a middle stage, such as the cusp‐calcifying stage, were visualized as the imperfect‐tubular form, similar to the maxillary accessional tooth forms (Figure 2(i)–(l)). In mandibular third molars in cusp‐calcifying periods, GTs were visualized as tubular forms like the maxillary third molars (Figure 2(m)–(p)). However, the GTs took a Y‐shape, not a J‐shape. The structures consisted of two forms of penetrating area. One was directed mesially as a parallel part, and the other directed vertically to the alveolar crest as a perpendicular part. In a late stage, such as the whole crown calcified stage of mandibular first, second, and third molars, GTs were visualized as hole forms, like the maxillary accessional teeth (Figure 2(q)–(t)). The only case without GTs was detected in the mandibular third molar, as a tooth bud completely surrounded by bone.

Detection ratios for GTs in the mandibular accessional teeth of subjects are also shown in Table 2.

**FIGURE 3:**
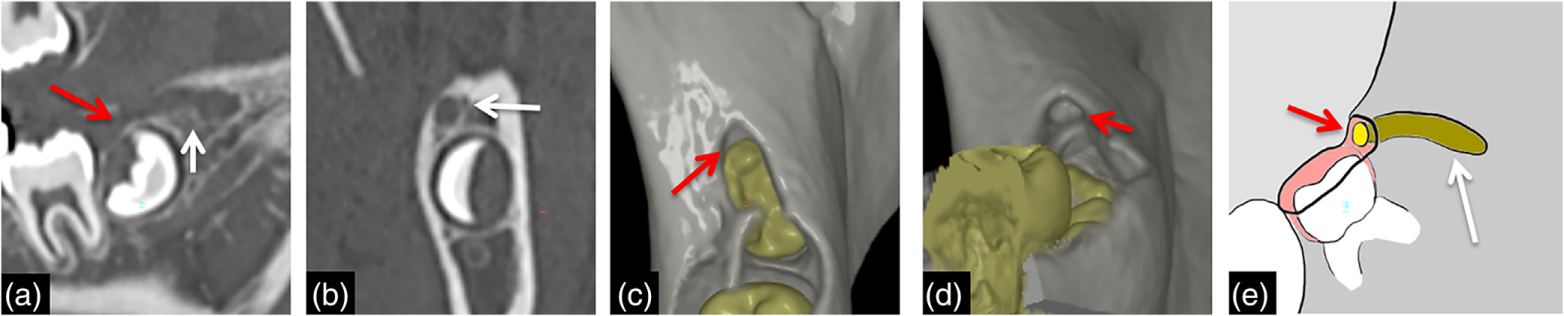
Representative images of the tract continuing from mandibular third molar bud to distal side. Representative sagittal (a) and coronal (b) images, 3D‐CT image (c, d) and schema (d) of the tract continuing from mandibular third molar bud to distal side. GTs are visualized as bone defect lines (arrows) on the distal alveolar crest continuous from the top of the mesial dental sac (red arrows)

Around mandibular third molars, GTs showed very interesting characteristics. Tracts continuing from the distal side of mandibular third molars were detected in many cases (Figure 3; Table 3). However, the detection ratio for characteristic tracts increased until 14 years old, but decreased thereafter. After 18 years old, the detection ratio for characteristic tracts was 0%.

**TABLE 3:** Detection ratio of tracts continuing from distal side of mandibular third molar in cases with third molars

	Number of cases		
Age	Detected	Not detected	Detection ratio (%)
1–7	–	61	0.0
8	2	30	6.3
9	7	41	14.6
10	4	31	11.4
11	1	18	5.3
12	9	9	50.0
13	18	21	46.2
14	10	6	62.5
15	4	15	21.1
16	–	5	0.0
17	3	8	27.3
18–30	–	18	0.0
Total	58	263	18.1

### 3.3 Alteration of GTs in accessional teeth on CT with aging

**FIGURE 4:**
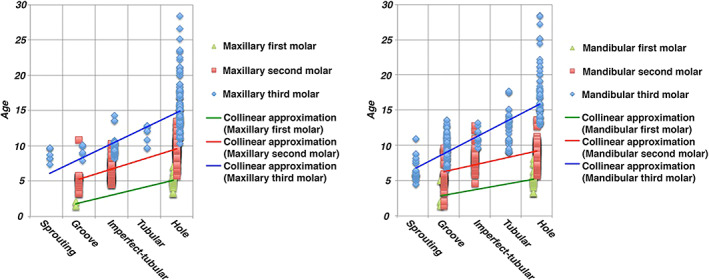
Relationship between each form of GT in maxillary and mandibular accessional teeth and age. Moderate to strong correlations are found between each form of GT and age (maxillary first molar: Spearman correlation *r* = 0.511, *p* < 0.001; maxillary second molar: Spearman correlation *r* = 0.717, *p* < 0.001; maxillary third molar: Spearman correlation *r* = 0.804, *p* < 0.001; mandibular first molar: Spearman correlation *r* = 0.364, *p* < 0.013; mandibular second molar: Spearman correlation *r* = 0.553, *p* < 0.001; mandibular third molar: Spearman correlation *r* = 0.863, *p* < 0.001)

Distributions of each form of GT and mean age are shown in Table 2. In maxillary or mandibular first, second, and third molars, findings of GTs in the teeth changed with age. The progression of forms was first the sprouting form, followed over time by the groove form, imperfect‐tubular form, tubular form, and finally hole form. However, no sprouting form was seen for maxillary and mandibular first and second molars, no imperfect‐tubular form was seen for maxillary and mandibular first molars, and no tubular form was seen for maxillary and mandibular first and second molars. In addition, no GTs were identified continuing from the distal side of the maxillary third molars. Variations of GTs for each accessional tooth and age range are shown in Figure 4. A correlation was seen between advanced alterations of GTs and age (maxillary first molar: Spearman correlation *r* = 0.511, *p* < 0.001; maxillary second molar: *r* = 0.717, *p* < 0.001; maxillary third molar: *r* = 0.804, *p* < 0.001; mandibular first molar: *r* = 0.364, *p* < 0.013; mandibular second molar: *r* = 0.553, *p* < 0.001; mandibular third molar: *r* = 0.863, *p* < 0.001).

### 3.4 Correlation between forms of GTs and formations of accessional teeth

Relationships between distributions of each GT form and formations of accessional teeth are shown in Table 4. A strong correlation was identified between advanced alterations of GTs and proportions of tooth development (maxillary: *r* = 0.623, *p* < 0.01; mandibular: *r* = 0.759, *p* < 0.01).

**TABLE 4:** Distribution of each form of GT and formation of accessional tooth

	Formation of accessional teeth																				
	First molar							Second molar							Third molar						
Form of GT	Not calcified	Cusp calcifying	Crown calcifying	Crown formation completed	Root developing	Root apex not closed	Root formation completed	Not calcified	Cusp calcifying	Crown calcifying	Crown formation completed	Root developing	Root apex not closed	Root formation completed	Not calcified	Cusp calcifying	Crown calcifying	Crown formation completed	Root developing	Root apex not closed	Root formation completed
Maxillary																					
Sprouting	–	–	–	–	–	–	–	–	–	–	–	–	–	–	7	–	–	–	–	–	–
Groove	–	–	4	–	–	–	–	5	14	8	–	–	–	–	5	1	–	–	–	–	–
Imperfect‐tubular	–	–	–	–	–	–	–	–	45	20	6	–	–	–	4	54	2	3	–	–	–
Tubular	–	–	–	–	–	–	–	–	–	–	–	–	–	–	–	8	1	–	–	–	–
Hole	–	–	14	17	15	‐	‐	‐	9	30	51	27	‐	‐	5	34	35	32	16	8	6
Not detected	–	–	–	–	–	–	–	–	–	–	–	–	–	–	–	1	1	1	1	–	–
Total	–	–	18	17	15	–	–	5	68	58	57	27	–	–	21	98	39	36	17	8	6
Mandibular																					
Sprouting	–	–	–	–	–	–	–	–	–	–	–	–	–	–	42	1	–	–	–	–	–
Groove	–	–	4	2	–	–	–	11	33	6	5	6	–	–	65	58	6	–	–	–	–
Imperfect‐tubular	–	–	–	–	–	–	–	–	13	22	8	6	2	–	1	14	1	–	–	–	–
Tubular	–	–	–	–	–	–	–	–	–	–	–	–	–	–	5	17	7	14	6	–	–
Hole	–	–	13	13	12	2	–	–	7	26	31	24	4	3	–	4	16	36	13	6	8
Not detected	–	–	–	–	–	–	–	–	–	–	–	–	–	–	–	1	–	–	–	–	–
Total	–	–	17	15	12	2	–	11	53	54	44	36	6	3	113	95	30	50	19	6	8

## 4 DISCUSSION

One of the most interesting results of the present study was the demonstration of characteristic appearances of GTs for accessional teeth. Various forms of GTs were visualized at the stages in maxillary and mandibular third molars. Based on the appearances of GTs in maxillary and mandibular third molars, forms were termed “sprouting form,” “groove form,” “imperfect‐tubular form,” “tubular form,” and “hole form.” In addition, GTs in teeth changed with aging and the proportion of calcification of the crown. Some forms of GTs were also visualized in first and second molars. Forms with aging followed the progression from the sprouting form to the groove form, imperfect‐tubular form, tubular form, and finally hole form. These various findings of GTs in accessional teeth were not similar to those in successional dentitions.

A possible explanation for the various findings of GTs of accessional teeth with aging is described below. Basically, dental lamina may first sprout from the distal bony wall of the dental sac of the mesial tooth bud. In the development of the molar bud, the alveolar crest around the molar bud and its dental lamina temporarily disappears, and a groove shape appears continuous from the molar bud to the mesial alveolar crest. The palatal (lingual) and buccal bone of the dental lamina develops and progresses to the imperfect‐tubular form. In the next phase, the bony wall on the alveolar crest of the dental lamina appears and forms a tubular shape as a GT. As a result, the GT forms a tubular J‐shape in the maxilla or a Y‐shape in the mandible. Regarding the Y‐shape in the mandible, even in the calcification phase of the bony wall on the parallel part, no bony wall is observed right over the perpendicular parts. In the next phase, the penetrating area in the maxilla changes direction from parallel to perpendicular. In the mandible, the penetrating area of the parallel part extends and fuses with the penetrating area of the perpendicular part. The direction of the penetrating area of the GT then changes from parallel to perpendicular. Regarding the first and second molars, however, because the alveolar crest bony walls of GTs would be more immature and less calcified in younger children, they could not be distinguished on CT. In addition, the sprouting form of GTs was not detected in first and second molars. A possible explanation is that the period visualized as the sprouting form for the first and second molars may occur before 1 year old (Nanci, 2013).

The shape of GTs closely resembles the 3D figures of dental lamina in textbooks of oral histology (Nanci, 2013). To the best of our knowledge, few precise descriptions have been reported for findings of GTs in accessional teeth, even in textbooks. A possible explanation is that the majority of dentists are interested only in tooth‐ or alveolar bone‐related diseases, not normal structures such as GTs. This is the first report to show that variations of GTs in accessional teeth and alterations according to aging could be identified and may be very significant for the eruption of accessional teeth. Many researchers have recently paid attention to the GTs of teeth from our previous reports (Gaeta‐Araujo et al., 2019; Koc et al., 2019; Nishida et al., 2015; Oda et al., 2018; Oda, Miyamoto, et al., 2016; Oda, Nishida, et al., 2016). Those studies elucidated that the lower detection ratio, abnormality of the attachment site, and the course and pathological status of GTs related to abnormal eruption (Gaeta‐Araujo et al., 2019; Koc et al., 2019; Nishida et al., 2015; Oda et al., 2018; Oda, Miyamoto, et al., 2016; Oda, Nishida, et al., 2016). In the present study, accessional teeth in which GTs were not detected only represented some third molars with higher mean age. We considered that one reason for this result was that some third molars tended to be impacted, while all first and second molars tended to erupt normally according to the patent fact of the difference in the frequency of impaction. Those previous reports focused on the detection ratio and attachment site of GTs, not the course and normal alterations of GTs in accessional teeth (Gaeta‐Araujo et al., 2019; Koc et al., 2019; Nishida et al., 2015). The fact that the shapes of GTs in the molars alter is very important for analyses in this field of study. For example, explanation of why mandibular third molars tend to be mesially inclined are based on the present appearance of GTs for mandibular third molars. The present findings indicate that dentists should pay more attention to the GT, and we expect that many more reports on these structures will be published in the future.

In addition, the present data might provide clues for common sites of odontogenic masses in the mandibular posterior region. One of the present findings was the existence of GT‐like structures extending from the distal side of mandibular third molars. We first named these GT‐like structures “pseudo‐GTs.” No mandibular fourth molar was detected in this study. Conversely, the structures seemed to disappear at a later phase. We speculated that the contents of the pseudo‐GT might represent dental lamina extending posteriorly. Previous studies have reported that remnants of the gubernacular cord in the GT could form the basis for the development of some odontogenic tumors and/or cysts (Bump, 1927; de Sa Cavalcante et al., 2018; Ide et al., 2011; Oda et al., 2018; Oda, Miyamoto, et al., 2016; Philipsen et al., 1992; Toller, 1967). If odontogenic masses are derived from pseudo‐GT, this would be one reason why common sites of odontogenic masses would be in the posterior mandibular region. This is the first report to describe this structure from CT images. The structure also very closely resembled the 3D appearance of dental lamina as described in oral histology‐related textbooks (Nanci, 2013).

A number of limitations should be considered for the present study. First, the numbers of maxillary and mandibular first molars were relatively small, because unerupted teeth could be visualized in only a small proportion of CT images. In addition, “sprouting‐form” GTs could not be visualized for first and second molars, possibly because no data were obtained from individuals under 1 year old. Second, we selected CT examinations that contained no abnormal findings from accessional teeth, for the purpose of uncovering normal alterations to the GTs of accessional teeth. Our data were only obtained from a relatively small cohort from a single institution. The present study of the relationship between aging and alteration of GT might not necessarily be correct, due to the retrospective nature of the study. The possibility that the correlation between change in GT form and age cannot be denied may actually be due to each individual characteristic of the GT. A further limitation of this study was that only Japanese subjects were included.

The present study described the imaging peculiarities and the process of progression of the molars as accessional teeth. Moreover, the existence of a GT‐like structure that we first named “pseudo‐GT” is shown. These preliminary data should facilitate the precise analysis of studies focused on the GTs of molars in the future.

## CONFLICT OF INTEREST

The authors have no conflicts of interest relevant to this article to report.

## AUTHOR CONTRIBUTIONS

M.O. conceptualized and drafted the initial manuscript; I.N., D.Y., and M.S. designed the study, critically reviewed the manuscript; M.H., O.T., S.T., H.T., T.O., and T.S. participated in the performance of the research, data collection critically reviewed the manuscript; T.T. and N.W. participated in the performance of the research, participated in data analysis, visualized the CT images, and critically reviewed the manuscript; S.M. participated in the performance of the research, data collection, using software for visualizing; Y.M. administrated the project, reviewed and edited the manuscript. All authors read and approved the final manuscript.

## ETHICS STATEMENTS

The present study was approved by the institutional review board of Kyushu Dental University (approval no. 14‐29). The need to obtain informed consent for participation in this trial was waived by the review board based on the retrospective design of the study.

## Data Availability

Data available on request due to privacy/ethical restrictions.
